# Supplementary material for: Study on measurement and prediction of agricultural product supply chain resilience based on improved EW-TOPSIS and GM (1,1)-Markov models under public emergencies
Source: PLoS One. 2025 May 9;20(5):e0321248. doi: 10.1371/journal.pone.0321248 (PMC12063874; doi:10.1371/journal.pone.0321248)
Supplement: Attachment — Submitted filename: Response to Reviewer2.docx [file pone.0321248.s002.docx]

**Response to the Reviewer 2 Comments**

**Dear Reviewer 2:**

**Thank you for your letter and for the reviewers’ comments concerning our manuscript entitled “Study on measurement and prediction of agricultural product supply chain resilience based on improved EW-TOPSIS and GM (1,1)-Markov models under public emergencies (ID: PONE-D-24-26636)”. Those comments are all valuable and very helpful for revising and improving our paper. We have tried our best to study comments carefully and made some changes in the manuscript. The reviewer comments are laid out below in italicized font and specific concerns have been numbered. Our response is given in normal font and changes are to the revised manuscript are given in the yellow text.**

***Q1.*** *The research gap and contribution are not clear in the Abstract. What problem did you study, and why is it important? The authors just mentioned the general issues in the supply chain. What is the main problem of the case study?*

**Response:** Thank you for your valuable feedback. We have revised the abstract accordingly, and in the resubmitted manuscript (Page 3, Lines 37-55), we have clearly stated that the core issue of this study is the measurement and development trends of the resilience of China’s agricultural product supply chain, and we have analyzed why this core topic is important. The main issues addressed in the case study are: Firstly, based on the case backgrounds of five leading agricultural product listed companies in China, we applied an improved EW-TOPSIS model to analyze the main factors affecting the development of agricultural product supply chain resilience during the COVID-19 pandemic. Secondly, we used an improved GM (1,1)-Markov model to predict the main factors influencing agricultural product supply chain resilience over the next five years. We ensured that the empirical analysis results are clearly and reasonably understood within the research context, providing insights into the resilience of agricultural product supply chains in other countries around the world.

***Q2.*** *An updated and complete literature review should be conducted to present the state-of-the-art and knowledge gaps in the research with strong relevance to the paper's topic. Some recent leading works have focused on optimizing the supply chain using optimization approaches such as metaheuristic approaches;*

**Response:** Thank you for your valuable suggestions. We have revised the literature review accordingly. In the resubmitted manuscript (from line 133 on page 7 to line 152 on page 8), we have discussed the application of metaheuristic methods in supply chains and added relevant references [7] to [16] to better highlight the research gaps and innovations of this dissertation. Additionally, we have compiled and summarized the methods related to the measurement, optimization, and prediction of agricultural product supply chains by various scholars, as shown in Table 1. The specific revisions have been highlighted in the literature review section.

***Q3.*** *I recommend extending the background on optimization methods, providing a thorough justification for the choice of method, and conducting a comprehensive sensitivity analysis using recommended approaches;*

**Response:** Thank you for your valuable feedback. We have introduced the background of using the improved EW-TOPSIS method and the improved GM (1,1)-Markov method in lines 233-240 on page 12. On one hand, the specific reasons for choosing the improved EW-TOPSIS method are detailed in lines 302-320 on pages 15-16, and the optimization steps are explained. On the other hand, the selection of the improved GM (1,1)-Markov method and its application in this study are described in lines 341-355 on pages 17-18. Additionally, a comprehensive sensitivity analysis of the two methods is conducted in Section 4.5 on page 35, line 642, to verify their feasibility.

This dissertation, using the Solomon case, evaluates the impact of inventory turnover rate (ITR), supply chain operating cost (SCOC), and supplier spatial distance (SSD) on supply chain resilience through sensitivity analysis.

Firstly, Sensitivity Analysis of Inventory Turnover Rate. This dissertation takes the top five agricultural product listed companies in China as examples, setting the inventory turnover frequency to 10, 15, 20, and 25 times respectively, running formula (5) to conduct sensitivity experiment tests, and analyzing the impact of inventory turnover rate on the resilience of the agricultural product supply chain. The results are compared as shown in Figure 5. The comprehensive case results reveal that as the supplier’s inventory turnover rate continues to increase, the resilience of the supply chain also continues to improve; when the supplier’s inventory turnover rate changes from 5 times to 10 times, the improvement in supply chain resilience is relatively small; when the supplier’s inventory turnover rate changes from 15 times to 20 times, and from 20 times to 25 times, the improvement in supply chain resilience is more significant. The analysis suggests that a high inventory turnover rate means that suppliers can quickly respond to market changes and order demands. Especially under public emergencies, this rapid response capability is an important indicator of supply chain resilience, as it allows suppliers to quickly adjust in the face of market fluctuations, thereby reducing the risk of supply chain disruptions. By analyzing the data from different cases, it is found that due to the relatively dispersed distribution of NHL’s suppliers, while the other four agricultural product listed companies have a more concentrated distribution of suppliers, when the inventory turnover rate of agricultural products continuously improves, the increase in the resilience of NHL’s supply chain is more significant. In contrast, the increase in the resilience of the supply chains of SQF, DBN, YILI, and HTGF is relatively smaller.

Secondly, conducting sensitivity analysis of supply chain operating cost. In this study, the operating costs of the agricultural product supply chain were set at 100,000 yuan/year, 150,000 yuan/year, 200,000 yuan/year, and 250,000 yuan/year, respectively. Using formula (6), sensitivity experiments were conducted to analyze the impact of agricultural product suppliers’ operating costs on the resilience of the supply chain. From the comprehensive case study results, it can be observed that as supply chain operating costs continue to rise, supply chain resilience decreases. When supply chain operating costs increased from 50,000 yuan/year to 100,000 yuan/year, the decline in supply chain resilience was significant; whereas, when costs rose from 150,000 yuan/year to 200,000 yuan/year, and from 200,000 yuan/year to 250,000 yuan/year, the decrease in resilience was less pronounced. The reasons for this include: On one hand, during the COVID-19 pandemic, lockdown measures and border closures in various countries and regions led to logistical disruptions and a significant increase in transportation costs. Delays and increased expenses in air and sea transportation, as well as traffic controls for land transport, made the transportation of agricultural products more difficult and expensive. To address these challenges, listed agricultural companies had to bear higher transportation costs, which not only increased operating costs but also reduced the flexibility and responsiveness of the supply chain. On the other hand, the uncertainty of market demand in the post-pandemic period has increased. To cope with sudden changes in supply and demand, companies had to increase inventory levels, leading to higher storage and management costs. However, excessive inventory not only ties up a large amount of capital but can also lead to waste due to the shelf-life limitations of agricultural products. The increased cost of inventory management further weakened the financial condition of enterprises. Therefore, as the investment in supply chain operating costs increases, supply chain resilience is further reduced.

At last, conducting sensitivity analysis of supplier spatial distance. This dissertation selects five agricultural product listed companies and sets the spatial distances of agricultural product suppliers at 10km, 15km, 20km, and 25km respectively. By running formula (7), sensitivity experiments are conducted to analyze the impact of supplier spatial distance on the resilience of the supply chain. As shown in Figure 7, with the increasing spatial distance of agricultural product suppliers, the resilience of the supply chain continues to decline. When the supplier’s spatial distance changes from 10 kilometers to 15 kilometers, the decrease in supply chain resilience is relatively small. However, when the supplier’s spatial distance changes from 15 kilometers to 20 kilometers, and then from 20 kilometers to 25 kilometers, the decline in supply chain resilience is more significant. The analysis suggests the following reasons: on one hand, the increase in the supplier’s spatial distance lengthens the transportation time, resulting in a longer transportation cycle for agricultural products from the production site to the consumption site. This not only increases logistics costs but also reduces the response speed of the supply chain. On the other hand, the increase in distance leads to more supply chain nodes (such as transfer warehouses, logistics hubs, etc.), and each node can potentially become a bottleneck or risk point. Therefore, a larger supplier spatial distance is detrimental to the improvement of supply chain resilience.

***Q4.*** *A robust and comprehensive section is necessary for their Data Collection. How do authors collect their data? What are the sources of data;*

**Response:** Thank you for your valuable feedback. We have introduced the data source and processing procedures of this article from line 286 on page 14 to line 300 on page 15. On the one hand, the objective data of this dissertation mainly comes from the CSMAR database and the annual reports of agricultural product listed companies. On the other hand, the main data indicators are mainly collected through questionnaires, involving managers of listed companies, professionals in the industry, and scholars in related fields. They score the performance of the samples under various subjective indicators, with a total score ranging from 1 to 5. The final value of each record is the average score of the questionnaire. We have also explained the reliability and accuracy of both subjective and objective data. The specific modifications have been highlighted in the data collection section. In addition, we have provided the raw data for this article in the attachment.

***Q5.*** *A detailed explanation is required for the case study—the location of suppliers, the distances, type of transportation, unit cost for transport, holding, storage, and so on;*

**Response:** Thank you for your insightful comments. We have added a 3.2 Case study section on page 12, line 241, which details five agricultural product listed companies from aspects such as supplier location, supply chain operating costs, and supplier spatial distance, and created Table 2. Based on this background, we have elaborately discussed the calculation steps of the Solomon sensitivity example to evaluate the impact of inventory turnover rate (ITR), supply chain operating cost (SCOC), and supplier spatial distance (SSD) on supply chain resilience. After determining the weights using the Improved Entropy-TOPSIS method, this dissertation combines Solomon’s examples to evaluate the impact of inventory turnover rate (ITR), supply chain operating cost (SCOC), and supplier spatial distance (SSD) on supply chain resilience through sensitivity analysis. The following steps can be taken to achieve this:

***Step 1:*** constructing a supply chain resilience evaluation function.

$R=W_{ITR}*ITR+W_{SCOC}*SCOC+W_{SSD}*SSD$ (1)

Where $W_{ITR}$ represents the weight of inventory turnover ratio, $W_{SCOC}$ represents the weight of supply chain operating cost, and $W_{SSD}$ represents the weight of supplier spatial distance. ITR, SCOC, and SSD indicate the standardized values.

***Step 2:*** Calculating the partial derivatives of supply chain resilience (R)with respect to each parameter to assess the impact of each parameter on supply chain resilience.

$\Delta R_{ITR}=R\left( ITR+\Delta ITR,SCOC,SSD \right)-R(ITR,SCOC,SSD)$	(2)

$\Delta R_{SCOC}=R\left( ITR,SCOC+\Delta SCOC,SSD \right)-R(ITR,SCOC,SSD)$	(3)

$\Delta R_{SSD}=R\left( ITR,SCOC,SSD+\Delta SSD \right)-R(ITR,SCOC,SSD)$ (4)

***Step 3:*** Calculating Sensitivity.

Inventory turnover rate sensitivity calculation formula:

$S_{ITR}=\frac{\Delta R_{ITR}}{\Delta ITR}$	(5)

Supply chain operating cost sensitivity calculation formula:

$S_{SCOC}=\frac{\Delta R_{SCOC}}{\Delta SCOC}$	(6)

Supplier spatial distance sensitivity calculation formula:

$S_{SSD}=\frac{\Delta R_{SSD}}{\Delta SSD}$	(7)

When $S_{ITR}$is large, it indicates that the inventory turnover rate has a significant impact on supply chain resilience. If $S_{SCOC}$ is large, it indicates that supply chain operating costs have a significant impact on supply chain resilience. If $S_{SSD}$ is large, it indicates that the supplier’s delayed delivery rate has a significant impact on supply chain resilience.

Through sensitivity analysis methods, the impact of supply chain growth parameters on supply chain resilience can be quantified, thereby providing decision-making support for the optimization of agricultural product supply chains.

***Q6.*** *Validation and verification are the main parts of the modeling approach. Please add a separate section to show the validation of the developed model;*

**Response:** Thank you for your valuable suggestion. We have added a new section 4.3, titled “Model verification and significance testing”, on page 30, line 548. On one hand, we conducted a residual test on the improved GM (1,1)-Markov method. The results show that, except for NHL, the majority of the samples passed the test. On the other hand, an independent samples t-test was performed, and the results indicate that, under most samples, the predicted value sequences of the two forecasting models passed the significance test. Moreover, the Markov correction model had a higher average level of significance, which further validates the rationality of the improved GM (1,1)-Markov method.

***Q7.*** *A separate strong discussion before the conclusion should be revised to discuss the results and compare them with similar studies. What are the managerial and* *practical implications of this work? How do the implemented approach and results help the selected case study? All details should be discussed and organized well;*

**Response:** Thank you very much for your profound insights. We have conducted a robust discussion and analysis of the empirical results in Section 4.6, Line 714, Page 39, where we compare them with similar studies, elucidate the management practice implications of this work, and explain how the implemented methods and results have contributed to the selected case study. The specific discussion is as follows:

Relevant research scholars have conducted studies on the probability of supply chain risks and the level of vulnerability, mainly focusing on rough measurement and prediction of supply chain resilience. Distinguished from existing studies (Yan et al., 2021; Qi et al., 2022), this dissertation constructs a supply chain resilience evaluation model tailored to the characteristics of the agricultural products industry by adopting an improved EW-TOPSIS. Based on the scientific construction of the evaluation index system, it effectively overcomes the unreasonable impact on index weights caused by over-concentrated or overly sparse sample data distribution under some indicators, obtaining reasonable weights for each index, and deriving the supply chain resilience scores of five leading listed companies in the agricultural products industry. Meanwhile, an improved Markov-modified GM (1,1) model is employed to address the poor prediction effect of traditional grey models for oscillating sequences, achieving high-precision fitting of the existing development trends of agricultural product supply chain resilience, and deriving relatively credible trend prediction values for the next five years. Therefore, compared with existing research results, this study can more accurately and reasonably measure and predict the resilience of agricultural product supply chains, providing reliable references for the overall development trends of China’s agricultural product supply chains.

Furthermore, the specific analysis is shown below:

First of all, an enterprise with a higher market capitalization, a large size, or strong profitability would not exhibit strong supply chain resilience. The results of the study showed that the ranking of market value is not consistent with the ranking of resilience level. For example, the NHL owed the highest market value in 2022 among all the industries but showed a significant lack of resilience during the pandemic(Q2). Secondly, in the evaluation system, indicators such as delayed delivery rate, suppliers’ cash flow ratio, and inventory turnover ratio have higher weightings, indicating their greater contribution to supply chain resilience; under the backdrop of public crisis events, enterprises need to seek communication and collaboration more, through more open and transparent information sharing and cooperation mechanisms to jointly face difficulties, and work together with member enterprises in the supply chain to enhance resilience and corporate strength(Q1). All the enterprises along the domestic agricultural supply chain have to cohesion through cooperation altogether to prevent and defuse potential public health emergencies in the future. Thirdly, in the prediction of empirical results, it was found that all five companies had their best performance in 2020; primarily because in 2020, the government’s support for ensuring supplies was strong, and a few companies with ample inventory (mainly leading listed companies) quickly utilized emergency logistics to sell their products; however, performance alone cannot fully indicate supply chain resilience, and in fact, the resilience scores of these companies in 2020 were relatively low, mainly due to the significant impact of negative indicators such as logistics efficiency and delayed deliveries, and the ability to quickly liquidate inventory does not entirely represent supply chain resilience. By predicting the development trend of agricultural product supply chain resilience for sample enterprises from 2023 to 2027, it shows that under the background of the weakening impact of the pandemic, the resilience of each enterprise is gradually and steadily improving (Q3). Finally, this research work combines the Solomon case study to evaluate the impact of inventory turnover rate (ITR), supply chain operating cost (SCOC), and supplier spatial distance (SSD) on supply chain resilience through sensitivity analysis. The case study analysis reveals that, under public emergencies, agricultural product listed companies should increase the inventory turnover rate, reduce supply chain operating costs, and shorten the spatial distance between suppliers, in order to better cope with demand fluctuations within each link of the supply chain.

The methods and results implemented in this article will provide guiding significance for the selected case study in the following aspects. Firstly, this research adopted an improved EW-TOPSIS method to construct an agricultural supply chain resilience evaluation system in line with the traits of the agri-food industry. The method overcame the unreasonable disturbance of overly dense or sparse distribution of sampled data, and derived the reasonable proportion of indicators, influence factors of the supply chain of which is selected by a scientific principle, and assessed the resilience level of five top listed agricultural companies. An improved Markov-modified GM (1,1) forecast model was established in this research, which overcame the deficiency of the predictive effect on oscillatory sequences and achieved a highly fitted effect of the development tendency of five sampled companies’ supply chain resilience level. Secondly, based on the research findings of evaluation and forecasting models in this dissertation, improvement mechanisms are proposed from multiple levels such as government, industry, and enterprises to effectively address the issues in the selected case studies (Q4):

(1) Government subsidy mechanisms. Government subsidies are an important factor in ensuring the stable operation of the agricultural product supply chain during emergencies. Among the various methods the government uses to regulate the macroeconomic context and ensure the smooth operation of major livelihood industries, economic subsidies are the most direct and effective. The government should proactively conduct grassroots research to understand the situation of enterprises in difficulty and formulate subsidy programs that comprehensively grasp the damage to enterprises immediately after the occurrence of emergencies. On one hand, accelerate the delivery of payments for government procurement in which enterprises participate, and appropriately extend or reduce the payment of taxes and social security funds by enterprises; on the other hand, encourage financial institutions such as state-owned banks to relax financing and loan limits for enterprises in distress, thus injecting funds into the market to boost market confidence and provide direct financial support for enterprise production.

(2) Development of digital supply chain mechanisms. In recent years, the digital transformation of large enterprises has been a significant topic in the field of enterprise management. Table 5 shows that the delayed delivery rate, supplier cash flow ratio, and inventory turnover rate account for 14.00%, 10.60%, and 8.00%, respectively. The construction and development of digital supply chains can directly influence the efficiency and effectiveness of inter-enterprise collaboration and enhance the transparency of supply chain information. As leading enterprises in the industry, five companies should take the initiative to play a leading and driving role in building digital supply chains, for example, YILI has a large number of suppliers and should actively construct an intelligent supplier model, focusing on timely control during the change period of suppliers, reducing the time for interest coordination between various departments in agriculture, and enhancing agriculture’s rapid response ability and regulatory capacity toward suppliers in major emergencies; DBN, with a large number of suppliers and relatively stable supply relationships, should effectively coordinate among suppliers in various forms, forming multi-channel partnerships and promoting information resource sharing and complementary advantages. SQF, maintaining its leading position in the agricultural sector as a listed company, needs to build a digital management system to coordinate internal management with external supply chain management to maintain market share and prevent potential crises of “large enterprise diseases”; HTGF and NHL should actively build digital supply chain management platforms, optimize internal and external management efficiency, and enhance decision-making and management capabilities using large-scale data.

(3) Innovation in multi-source procurement channel mechanisms. As shown in Table 6, the dimension of agricultural product supply chain regulation capability accounts for 31.10% of the evaluation index system, making it one of the most important dimensions determining the resilience of the agricultural product supply chain. Therefore, the agricultural product supply chain industry should comprehensively build diversified supply channels for agricultural enterprises, connecting offline and online networks to form a unified supply approach, strengthening the cooperative relationships with agricultural processing enterprises, and standardizing production environments, processing, and acceptance processes. NHL, primarily engaged in seed business, has a complete system for seed research, development, and sales, which provides a good condition for constructing self-cultivated and self-sold agricultural bases. It can expand internal procurement, turnover, and supply and delivery models, implementing intelligent control and integrating them comprehensively into enterprise management. SQF and HTGF, with a wide variety of product types and dispersed procurement sources, should establish their own procurement platforms, screen high-quality suppliers through online bidding channels, and periodically evaluate the performance of upstream enterprises in the supply chain, selecting high-quality enterprises for long-term cooperation, gradually perfecting a fixed multi-source procurement system; YILI and DBN can enhance cooperation and links with upstream enterprises and improve the resilience of the supply chain embodied in the procurement process by building online and offline supply networks, unifying procurement channels, and standardizing procurement procedures and logistics systems.

(4) Improvement of emergency response mechanisms for sudden events. Defining the work areas, service items, operating conditions, working methods, cooperation principles, and supervision aspects of various units helps to mitigate the risks brought about by sudden events. Although YILI and HTGF are large-scale enterprises, they exhibited insufficient resilience during the pandemic and should comprehensively consider the local characteristics, natural and geographical environments, production and personnel allocation, social productivity distribution, and actual transportation conditions of their subsidiaries in various locations. Under the guidance and support of governments at all levels, they should establish a three-level supply network with “district-town (street, committee)-community (village)” that is longitudinally connected, horizontally supported, and appropriately scaled; DBN, whose main suppliers are agricultural production cooperatives, is suitable for promoting digital reform in emergency materials in rural areas, establishing a “village-town (street)-district” three-level linkage mechanism. SQF and NHL should focus on emergency material digital platforms, improving coverage and inventory rates, and ensuring scientific, effective, and timely management of the entire process of material reserves, strengthening the allocation of materials for emergency response.

The management and practical significance of this work are as follows. On the one hand, this research will assist governments and stakeholders in other countries in understanding the key elements of supply chains and strengthening supply chain resilience. Identifying core elements can help managers find the strengths and weaknesses of supply chains. By focusing on relevant indicators, they will determine the effectiveness of existing supply chain management strategies. Additionally, they can learn from major public emergencies to establish mechanisms for pre-event prevention, in-event adjustment, and post-event feedback, thereby promoting the optimization and sustainable development of global agricultural product supply chains. On the other hand, this research identifies “agricultural product supply chain adjustment capabilities (A3)” as the key determinant to achieve resilience in agricultural product supply chains, warranting the highest priority. The COVID-19 pandemic has highlighted the importance of adjustment capabilities for agricultural product-listed companies in building resilient supply chains. The agricultural product supply chain faces numerous challenges, such as natural disasters, market fluctuations, policy changes, etc., which can all impact the supply chain. Supply chains with strong adjustment capabilities can manage inventory more effectively, reducing overstocking or stockouts. Therefore, in this context, adjustment capability is not only a theoretical concept but also a practical necessity for companies to survive and thrive in times of crisis, directly relating to the company’s profitability and market competitiveness. All stakeholders should pay attention to the effective coordination of various links in the agricultural product supply chain, such as delayed delivery rate (B12), supplier cash flow ratio (B7), inventory turnover rate (B6), etc. On one hand, enterprises upstream and downstream of the supply chain should build information-sharing mechanisms, such as data on market supply and demand, price fluctuations, and logistics status, to improve the overall efficiency of the supply chain. On the other hand, long-term strategic cooperative relationships should be established with suppliers and customers to ensure the stability and sustainability of the supply chain. A reasonable interest distribution mechanism should be formulated to incentivize participants at all links of the supply chain to jointly maintain its resilience.

***Q8.*** *What is this work's managerial and practical implication for the selected case study? How could these results help the governments and stakeholders in other countries;*

**Response:** Thank you for your valuable comments. We have discussed in detail the managerial implications of the research for the selected case studies, as well as how it can assist governments and stakeholders in other countries, in lines 838-863 on pages 44-45. This is also mentioned in the 7th issue above. The specific discussion is as follows:

The management and practical significance of this work are as follows. On the one hand, this research will assist governments and stakeholders in other countries in understanding the key elements of supply chains and strengthening supply chain resilience. Identifying core elements can help managers find the strengths and weaknesses of supply chains. By focusing on relevant indicators, they will determine the effectiveness of existing supply chain management strategies. Additionally, they can learn from major public emergencies to establish mechanisms for pre-event prevention, in-event adjustment, and post-event feedback, thereby promoting the optimization and sustainable development of global agricultural product supply chains. On the other hand, this research identifies “agricultural product supply chain adjustment capabilities (A3)” as the key determinant to achieve resilience in agricultural product supply chains, warranting the highest priority. The COVID-19 pandemic has highlighted the importance of adjustment capabilities for agricultural product-listed companies in building resilient supply chains. The agricultural product supply chain faces numerous challenges, such as natural disasters, market fluctuations, policy changes, etc., which can all impact the supply chain. Supply chains with strong adjustment capabilities can manage inventory more effectively, reducing overstocking or stockouts. Therefore, in this context, adjustment capability is not only a theoretical concept but also a practical necessity for companies to survive and thrive in times of crisis, directly relating to the company’s profitability and market competitiveness. All stakeholders should pay attention to the effective coordination of various links in the agricultural product supply chain, such as delayed delivery rate (B12), supplier cash flow ratio (B7), inventory turnover rate (B6), etc. On one hand, enterprises upstream and downstream of the supply chain should build information-sharing mechanisms, such as data on market supply and demand, price fluctuations, and logistics status, to improve the overall efficiency of the supply chain. On the other hand, long-term strategic cooperative relationships should be established with suppliers and customers to ensure the stability and sustainability of the supply chain. A reasonable interest distribution mechanism should be formulated to incentivize participants at all links of the supply chain to jointly maintain its resilience.

***Q9.*** *What is the* *generalizability aspect of this work compared to other similar SC worldwide;*

**Response:** Thank you for your valuable feedback. We have clearly pointed out on pages 39, lines 715 to 40, lines 730, that compared to similar supply chain studies globally, the universality of this study is stronger, with specific discussions as follows:

Compared to other similar SC worldwide (Yan et al., 2021; Qi et al., 2022), scholars have conducted research on the probability of supply chain risk occurrence and the degree of vulnerability, mainly focusing on imprecise measurement and prediction of supply chain resilience. However, due to the high sensitivity of agricultural product supply chains to internal and external environmental changes, comprehensive evaluation and long-term trend forecasting of supply chain resilience in a more complex external environment are relatively scarce. Therefore, this dissertation adopts an improved EW-TOPSIS method to construct an evaluation model for the resilience of agricultural product supply chains. Based on the scientific construction of an evaluation indicator system, it effectively overcomes the unreasonable impact of overly dense or sparse sample data distribution under certain indicators on the indicator weights, obtaining reasonable weights for each indicator, and deriving the supply chain resilience scores of five leading listed companies in the agricultural products sector. At the same time, an improved Markov-modified GM (1,1) model is used to overcome the shortcomings of traditional gray models in predicting oscillatory sequences, achieving high-precision fitting of the current development trends of agricultural product supply chain resilience, and deriving relatively credible predictions for the next five years. Therefore, compared to existing research findings, this study can more accurately and reasonably measure and predict the resilience of agricultural product supply chains, providing a reliable reference for the overall development trends of domestic agricultural product supply chains, and its applicability is stronger.

***Q10.*** *The conclusion is very general. The conclusion should be revised and improved by adding this work's significant contribution, results, and limitations;*

**Response:** Thank you for your valuable feedback. We have revised and improved the conclusion section from page 45, line 865 to page 48, line 931, adding important contributions, results, and limitations of this study. The specific solutions are as follows:

Firstly, the main contributions of this dissertation r are as follows: This article is based on the research background of public emergencies. On the one hand, five typical agricultural product listed companies from 2015 to 2022 were selected as research samples. This article constructed an agricultural product supply chain resilience index system covering four dimensions: receptivity, adaptability, resistance, and recovery, and used an improved EW-TOPSIS model to scientifically measure the resilience of their agricultural product supply chains. On the other hand, the improved GM (1,1)-Markov model proposed in this research work was used to accurately predict the development trend of the agricultural product supply chain resilience of the five agricultural product companies from 2023 to 2027.

Secondly, the main findings of this study are as follows: (1) This dissertation divides the evaluation indicators of agricultural product supply chain resilience from both quantitative and qualitative perspectives, establishing a more complete evaluation system for the supply chain resilience of agricultural enterprises. In the improved entropy weight-TOPSIS model, the introduction of Z-score parameters effectively overcame the unreasonable impact on the weight of indicators caused by the overly dense or sparse distribution of sample data under some indicators, obtained reasonable weights for each indicator, and derived the supply chain resilience scores of the top five agricultural product listed companies, improving the accuracy of the evaluation results. Through empirical analysis, the primary influencing factors of agricultural product supply chain resilience were identified, explaining the importance of indicators such as the delayed delivery rate, supplier cash flow ratio, and inventory turnover rate for enterprise supply chain resilience(Q1); it also demonstrated a general trend where the level of agricultural product supply chain resilience declined in 2020 and 2021 and recovered and reached its highest point in 2022(Q2).(2) using the top agricultural product listed companies before and after the outbreak of the COVID-19 pandemic over eight years as samples, a resilience prediction model was constructed using the improved GM (1,1)-Markov prediction method to predict the development trends in the resilience of the agricultural product supply chains of the sample companies from 2023 to 2027. It presented a trend of gradual and stable improvement in resilience as the impact of the COVID-19 pandemic gradually diminishes(Q3). The resilience prediction model proposed in this study can predict changes in resilience indicators in real-time, providing assistance in the evaluation and early warning of supply chain resilience of agricultural product listed companies, thereby reducing the risk of chain breakage and blockage in uncertain environments. However, the limitation of this study lies in its dependence on the collection of financial indicator data from agricultural product listed companies. Therefore, future research should focus on acquiring the required data through multiple channels. (3) The prediction effectiveness of the resilience prediction model needs to be tested for significance. By conducting independent samples T-tests on the predicted series and the original series, the mean difference was compared to determine if it was significant. The results found that at the significant level, there was no significant difference between the GM (1,1) prediction series and the Markov corrected series; the prediction effects of the five agricultural product listed companies passed the significance test with high significance and small mean differences, thereby verifying the feasibility of the improved GM (1,1)-Markov. In addition, this dissertation combines the Solomon case study to evaluate the impact of inventory turnover rate (ITR), supply chain operating costs (SCOC), and supplier spatial distance (SSD) on supply chain resilience through sensitivity analysis. The results show that improving inventory turnover rate, reducing operating costs, and shortening supplier spatial distance are key measures to enhance supply chain resilience. Meanwhile, in combination with the case background of the five agricultural product listed companies and the results obtained from empirical research, five categories of agricultural product supply chain enhancement mechanisms are proposed at the government level, industry level, and enterprise level, aiming to provide reference for the future development of relevant fields(Q4).

At last, like other studies, this study has some limitations: (1) Errors could occur due to these data sources being major relevant to the financial performances of the sampled company, which enlarged the effect of finance on the resilience system. The data of objective quantitative indicators are collected from publicly disclosed annual financial reports of sample companies, which leads to a significant impact on the economic status of sample companies on supply chain resilience. (2) from the perspective of the model methods. The improved GM (1,1)-Markov model is significantly influenced by the scale of the data. while this research only gathered 15 indicators of 5 samples during 8 years, 600 records of data. This could result in a small scale of the state space in the Markov process and the forecast model could not completely stimulate the complexity of external or internal changes in the real environment.

***Q11.*** *The structure of paper (just 5 sections are needed) should be revised. Introduction-Methodology (data collection-case study)-Literature review- results and discussion- Conclusion (all sub-sections (6.1-6.3) should be transferred to the discussion part);*

**Response:** Thank you for your constructive feedback. We have revised the paper structure into five sections and have clearly marked the changes from page 6, line 116 to page 7, line 121, specifically categorized as: Introduction, Literature Review, Methodology, Results and Discussion, and Conclusion. All subsections from 6.1 to 6.3 have been transferred to the Discussion section. The specific revisions have been highlighted in the revised version of the dissertation.

Once again thank you much for your comments and suggestions, we tried our best to improve the manuscript and made some changes in the re-submit manuscript. We appreciate for your warm work earnestly, and hope the correction will meet with approval.

Looking forward to hearing from you soon.

Yours sincerely,

Li Lu on behalf all authors.
